# Occupational Exposure to HIV: Perceptions and Preventive Practices of Indian Nursing Students

**DOI:** 10.1155/2014/296148

**Published:** 2014-04-17

**Authors:** Siddharudha Shivalli

**Affiliations:** Community Medicine, Yenepoya Medical College, Yenepoya University, Mangalore 575018, India

## Abstract

*Introduction*. Nurses have a frontier caring role that brings them in close contact with patients' blood and body fluids. An understanding of their professional behavior is essential to assess and minimize the occupational exposure to HIV among them. *Objectives*. (1) To appraise the knowledge, attitudes, and preventive practices of nursing students pertaining to occupational exposure to HIV. (2) To quantify the risk and correlates of exposure to HIV among them. *Methodology*. Cross-sectional study was conducted in a nursing college of Varanasi, India. A semistructured and pretested pro forma consisting of questions pertaining to modes of HIV transmission, universal precaution practices, and various aspects of nursing HIV patients was utilized. Independent sample *t*- and *z*-tests were applied to judge the association of study variables with the knowledge and risk of HIV. *Results*. The study sample consisted of 87 female and 16 male nurses. Participants' knowledge of HIV transmission was satisfactory. More than 80% of them had an exposure to blood/body fluid in the last year. Exposure rates for blood/body fluid did not show a significant association (P > 0.05) with study variables. *Conclusion*. There were serious lacunae in implementation of the universal precautions despite satisfactory knowledge. Reinforcement of universal precautions is required.

## 1. Introduction

Nurses form the major chunk of various cadres of health care personnel in most of the countries. Their profession demands a frontline caring role bringing them in close contact with patients' blood and other body fluids. And this puts them at risk of occupational exposure to HIV/AIDS and other blood infections. According to the World Health Organization 2.5% of global HIV cases are due to occupational exposure among health care workers [1]. Such accidents are associated with a few, but pose significant risk to health care professionals' health, career, families and also to patients under their care [2].

Prevention of blood/body fluid exposure through safer practices, barrier precautions, safer needle devices, and other innovations are the best ways to prevent HIV and other blood borne/body fluid pathogens [3, 4]. Widespread renaissance of other infectious diseases, such as tuberculosis, has added a new dimension to the increase of occupational risks. In this regard occupationally acquired HIV poses greater psychosocial challenges to the nurse due to associated stigma and discrimination. Hence, an understanding of their professional behavior is essential to assess and minimize the occupational exposure to HIV among them. In this quest a study was undertaken with the following objectives.

## 2. Objectives

The objectives of this paper were as follows.

(1) To appraise knowledge, attitudes, and preventive practices of nursing students of Varanasi in India with regard to occupational exposure to HIV. (2) To quantify the risk and correlates of exposure to HIV among them.

## 3. Methodology

A cross-sectional study was conducted in a nursing college of Varanasi, Uttar Pradesh state in India, from October 2012 to November 2012. Estimated adult HIV prevalence in Uttar Pradesh is 0.09% and estimated number of annual new HIV infection among adults is 7,745 [5]. Being one of the tourist destinations, Varanasi district is considered as highly vulnerable in the state for HIV. Studied nursing college is attached to a tertiary care teaching hospital which is a regional centre for National AIDS control program and caters to high number of HIV patients.

Ethical clearance was obtained from Ethics Committee of Banaras Hindu University before the inception of study. A semistructured and pretested pro forma was utilized to assess the knowledge, attitude, and occupation risk of exposure to HIV infection among nursing students. After obtaining permission from the nursing college principal, the purpose of the study was explained to students. All the nursing students with a minimum of one-year clinical/hospital exposure were included in the study. Written informed consent was taken from all the study participants. Anonymity was maintained during data collection to ensure confidentiality and to enhance the participation rate. The pro forma consisted of questions pertaining to modes of HIV transmission, exposure to blood/body fluid in the last one year, attitudes and practices of universal precautions, and various aspects of nursing HIV patients. Data thus generated was analyzed using SPSS v 11. Frequency and proportions were used to express the results. Independent sample *t*- and *z*-tests were applied to judge the association of study variables with the knowledge and occupational risk of HIV. The level of statistical significance was set at *P* ≤ 0.05 (two sided).

## 4. Results

A total of 103 nursing students participated in this study. The study sample consisted of 87 (84.5%) female and 16 (14.5%) male nurses. Mean age of the nursing students was 20.2 ± 1.22 years and mean time spent in the hospital was 4.31 ± 0.84 hrs. (range: 3–8 hrs.). Almost 70% (*n* = 72) of them (50% of male and 73.6% of female) perceived as they are at high risk of occupational exposure to HIV. This gender-wise difference in the perception was not statistically significant. (*P* = 0.058) Almost all of them (96.1%) had nursed an HIV patient in the last year.

Knowledge of the nursing students was assessed by asking them about the various modes by which HIV is and is not transmitted. Majority of the correct responses were recorded for blood (99%), urine (87.4%), feces (89.3%), mosquito bite (96.1%), and sweat/tears (88.3%). Nearly half of the nursing students gave correct responses to cerebrospinal fluid (45.6%), breast milk (57.3%), and saliva (53.4%) as source of HIV transmission. Except for “breast milk” as a potential source of HIV transmission, their knowledge level did not differ significantly (*P* > 0.05) according to the perceived risk of exposure to HIV in nursing profession (Table 1).

Attitudes of nurses towards universal precautions and various aspects of nursing HIV patients were mixed (i.e., both positive and negative). According to 97.1% of the nursing students all inpatients should be tested for HIV. Similarly, 94.2% of them opined that all nurses should also be tested for HIV. However, only 87.4% of them were willing to get their HIV test done. Surprisingly more than one-third of them (38.8%) were against the continuation of job if a nurse is HIV positive. Almost all of them (99%) also stressed on universal precautions while nursing. For majority of them self-protection was priority to confidentiality of HIV status of the patient. There was no significant association (*P* > 0.05) between the attitudes of the nurses and their perceived risk of exposure to HIV in nursing profession (Table 1).

Although study participants expressed positive attitudes for universal precautions there were serious lacunae in implementation of the same. Except for “hand wash,” all other practices were completely overlooked (Table 2). Inapt implementation of universal precautions was reflected in higher rates of exposure to blood/body fluid among nursing students.

Almost 8 out of every 10 nursing students had an exposure to blood or body fluid during nursing, in the last one year (Table 2). One-fifth of such exposures had occurred while nursing HIV patients (Table 3). Exposure of intact skin to blood/body fluid was the most common type of exposure (64.1%) followed by needle prick (38.8%) and cut from sharp instrument (35%). Many of students had multiple exposures. Unfortunately, most of the exposures were not reported to higher authorities. Neither the perceived risk of occupational exposure to HIV nor the hours of nursing had significant association (*P* > 0.05) with the exposure rates among study participants.

## 5. Discussion

In order to prevent occupational exposure to HIV infections, capacity building of nursing students is imperative. Ensuring adequate knowledge of HIV transmission and hands on training during student phase could go a long way in averting exposure to HIV. Strict compliance for universal precautions and apt management of exposures are crucial in this regard. Though many infections are transmitted by blood/body fluid exposure, HIV needs a special mention. Lack of curative treatment and prevailing social stigma and discrimination would always keep HIV in the lime light. In addition to devastating the familial, social, and economic lives of individuals, HIV stigma is cited as a major barrier to accessing prevention, care, and treatment services [6–8].

This study intended to explore the knowledge level, perception, and preventive practices of nursing students about occupational exposure to HIV. According to the World Health Organization, among various cadres of healthcare personnel, nurses are at maximum risk of getting exposed to HIV and other blood/body fluid borne infections. The same was perceived by the nursing students in our study and reports of the other studies [9–11].

Results revealed satisfactory knowledge of HIV transmission among the study participants and knowledge level did not vary significantly according to perceived risk of exposure to HIV in nursing (*P* > 0.05). Suboptimal knowledge regarding transmission of HIV/blood borne pathogens is reported by Hentgen et al. [9] and Jain et al. [12].

Almost all (97.1%) the nursing students wanted HIV testing for every inpatient. Execution of discriminatory measures of safety precautions based on the serostatus of the individual could be the rationale for such opinion. This attitude was reflected in their practices as the number of accidental exposures to blood/body fluid reduced drastically (to fifth) while nursing HIV patients.

This is corroborated by findings of Jain et al. [12] and Yamini et al. [13] who reported that HIV status of the patient was an important factor in the implementation of standard precautions. Such biased safety practices should be discouraged as HIV test negative patients may be in window period but can transmit the disease.

Unusual attitudes such as “isolation of HIV patients in quarantine”, “discomfort for use of gloves” and assuming that “reporting of accidental exposure is not important” were reported by Hentgen et al. [9], Le Pont et al. [14], and Shriyan et al. [15] in their studies among healthcare workers, respectively.

Unanimous opinion of study participants for periodic testing of all nurses for HIV is admirable. Such surveillance would help in early detection of seroconversion in unnoticed or unreported accidental exposures. However, the opinion of discontinuing nursing (38.8%) if a nurse is HIV positive is unethical and not endorsed. It must be emphasized that illness due to blood borne pathogens such as HIV, HBV, and HCV and TB infection is not a cause for discontinuation of employment. HIV positive nurses should be allowed to work, provided they practice universal precautions for infection control [16]. The International Labour Organization (ILO) Code of Practice affirms that HIV infection and AIDS should be managed in the workplace like any other serious illness and workers should enjoy normal job security as long as they are medically fit [17].

Serious lacunae in universal precautions were reflected in high exposure rates (81.6%) among study participants in the last 12 months. Varying rates of accidental exposures were reported by Singru and Banerjee [11] (39.63%), Le Pont et al. [14] (75%), Tetali and Choudhury [18] (74.5%), and Mashoto et al. [19] (48%) in their studies across the globe. It calls for the capacity building of nursing students to change their attitudes and reinforcement of universal precaution practices in work settings.

Despite higher rates of exposure, severe underreporting of the events was observed in the present study. Many authors also have highlighted the underreporting of accidental exposures in their studies [15, 18, 20–22]. Reporting all types of exposures is essential and should be encouraged in order to ensure adequate follow-up, testing, and management of the affected healthcare worker. It is also an excellent feedback of implementation of universal precautions.

## 6. Conclusion

Although nursing students' knowledge on HIV transmission was satisfactory, there were serious lacunae in implementation of the universal precautions. There is a need of capacity building of nursing students to change their attitudes and reinforce universal precaution practices in work settings.

## Figures and Tables

**Table 1:** Correct knowledge of HIV transmission and attitudes of nursing students according to their perceived risk of exposure to HIV in nursing profession.

Study variable	Perceived risk of exposure to HIV			*P* value
	High (*n* = 72)	Not high (*n* = 31)	Total (*n* = 103)	
Correct knowledge of HIV transmission				
Blood	71 (98.6)	31 (100)	102 (99)	0.509
Semen/vaginal secretion	48 (66.7)	24 (77.4)	72 (69.9)	0.257
Cerebrospinal fluid	31 (43.1)	16 (51.6)	47 (45.6)	0.423
Breast milk	29 (40.3)	20 (64.5)	59 (57.3)	0.02^†^
Urine*	64 (88.9)	26 (83.9)	90 (87.4)	0.483
Feces*	65 (90.3)	27 (87.1)	92 (89.3)	0.631
Sweat/tears*	65 (90.3)	26 (83.9)	91 (88.3)	0.352
Saliva*	42 (58.3)	13 (41.9)	55 (53.4)	0.126
Mosquito bite*	69 (95.8)	30 (96.8)	99 (96.1)	0.818
Attitudes of nursing students (response: I agree)				
All inpatients should be tested for HIV	69 (95.8)	31 (100)	100 (97.1)	0.250
Universal precautions are to be applied for all patients	71 (98.6)	31 (100)	102 (99)	0.502
Self-protection is more important than confidentiality of HIV status of the patient	59 (81.9)	25 (80.6)	84 (81.6)	0.872
HIV positive nurse should discontinue nursing job	26 (36.1)	14 (45.2)	40 (38.8)	0.39
All nurses should be tested for HIV	67 (93.1)	30 (96.8)	97 (94.2)	0.46
Willing to get my HIV test done	63 (87.5)	27 (87.1)	90 (87.4)	0.952

*not a potential source/route of HIV transmission was considered as correct response.

^†^Significant (*P* < 0.05).

**Table 2:** Exposure to blood/body fluid and Universal Precaution practices among nursing students according to their perceived risk of exposure to HIV in nursing profession.

Study variable	Perceived risk of exposure to HIV			*P* value
	High	Not high	Total	
	(*n* = 72)	(*n* = 31)	(*n* = 103)	
Universal precaution practices of nurses				
Wash hands while nursing	67 (93.1)	30 (96.8)	97 (94.2)	0.459
Always use gloves	34 (47.2)	12 (38.7)	46 (44.7)	0.425
Always use face mask	12 (16.7)	2 (6.5)	14 (13.6)	0.164
Always use gown	7 (9.7)	1 (3.2)	8 (7.8)	0.258
Always use eye protective goggle	3 (4.2)	1 (3.2)	4 (3.9)	0.818
Exposure to blood/body fluid				
Exposure to skin	46 (63.9)	20 (64.5)	66 (64.1)	0.952
Needle prick	25 (34.7)	15 (48.4)	40 (38.8)	0.190
Cut from a sharp	25 (34.7)	11 (35.5)	36 (35)	0.944
Exposure to mucosa	9 (12.5)	4 (12.9)	13 (2.6)	0.952
Any type of exposure	59 (81.9)	25 (80.6)	84 (81.6)	0.872

**Table 3:** Exposure to blood/body fluid among nursing students according to hours of nursing.

Exposure to blood/body fluid	Number (%) (*N* = 103)	Mean hours of nursing	SD	t	*P*
Nursing of all patients					
Exposed	84 (81.5)	4.37	0.861	1.491	0.139
Not exposed	19 (18.5)	4.05	0.71		
Nursing of HIV Positive patients					
Exposed	18 (17.5)	4.33	0.686	0.125	0.901
Not exposed	85 (82.5)	4.31	0.873		
